# *Escherichia coli* in Europe: An Overview

**DOI:** 10.3390/ijerph10126235

**Published:** 2013-11-25

**Authors:** Nerino Allocati, Michele Masulli, Mikhail F. Alexeyev, Carmine Di Ilio

**Affiliations:** 1 Department of Experimental and Clinical Sciences, “G. d’Annunzio” University of Chieti-Pescara, Chieti I-66013, Italy; E-Mails: masulli@unich.it (M.M.); carmine.diilio@unich.it (C.D.I.); 2 Department of Cell Biology and Neuroscience, University of South Alabama, Mobile, AL 36688, USA; E-Mail: malexeye@southalabama.edu

**Keywords:** *Escherichia coli*, Gram-negative bacteria, pathotype, multidrug resistance, epidemiology, phage therapy, antimicrobial peptides, combination therapy

## Abstract

*Escherichia coli* remains one of the most frequent causes of several common bacterial infections in humans and animals. *E. coli* is the prominent cause of enteritis, urinary tract infection, septicaemia and other clinical infections, such as neonatal meningitis. *E. coli* is also prominently associated with diarrhoea in pet and farm animals. The therapeutic treatment of *E. coli* infections is threatened by the emergence of antimicrobial resistance. The prevalence of multidrug-resistant *E. coli* strains is increasing worldwide principally due to the spread of mobile genetic elements, such as plasmids. The rise of multidrug-resistant strains of *E. coli* also occurs in Europe. Therefore, the spread of resistance in *E. coli* is an increasing public health concern in European countries. This paper summarizes the current status of *E. coli* strains clinically relevant in European countries. Furthermore, therapeutic interventions and strategies to prevent and control infections are presented and discussed. The article also provides an overview of the current knowledge concerning promising alternative therapies against *E. coli* diseases.

## 1. Introduction

*E. coli*, a member of the bacterial family of *Enterobacteriaceae*, is the most prevalent commensal inhabitant of the gastrointestinal tracts of humans and warm-blooded animals, as well as one of the most important pathogens [1]. As a commensal it lives in a mutually beneficial association with hosts, and rarely causes disease. It is, however, also one of the most common human and animal pathogens as it is responsible for a broad spectrum of diseases. The peculiar characteristics of the *E. coli*, such as ease of handling, availability of the complete genome sequence, and its ability to grow under both aerobic and anaerobic condition, makes it an important host organism in biotechnology. *E. coli* is used in a wide variety of applications both in the industrial and medical area and it is the most used microorganism in the field of recombinant DNA technology [2].

Prior to the identification of specific virulence factors in pathogenic strains, *E. coli* was principally classified on the basis of the serologic identification of O (lipopolysaccharide, LPS) and H (flagellar) antigens [1]. Based on the type of virulence factor present and host clinical symptoms, *E. coli* strains are classified into pathogenic types (pathotypes are defined as a group of strains of the same species causing a common disease): at least seven major pathotypes for enteric *E. coli*, whereas three *E. coli* pathotypes are extraintestinal strains (ExPEC) (Table 1) [1]. Intestinal pathogens spread through the faecal-oral route by ingestion of contaminated food or water.

EPEC strains cause diarrhoea primarily in children, particularly under conditions of poor hygiene, as well as in animals [1]. EHEC is a typically food-born pathogen causing haemorrhagic colitis or HUS [1]. Typical EHEC strains produce Shiga-like toxins (named Shiga toxin producing *E. coli*, STEC) similar to those produced by *Shigella dysenteriae* making them the most virulent diarrhoeagenic *E. coli* known to date [3]. ETEC are the most common pathogens causing travellers’ diarrhoea with mild to severe watery diarrhoea in humans of all ages [4,5]. EAEC strains are associated with persistent diarrhoea in humans, and have been recognized as the cause of several outbreaks of diarrhoeal disease worldwide. EAEC, frequently found in the gut of asymptomatic humans, is the second foremost cause of travellers’ diarrhoea worldwide. EAEC is frequently associated with diarrhoea in children in developing countries and in HIV-infected patients [6,7]. DAEC causes diarrhoea particularly in children [8]. EIEC is frequently cause of watery diarrhoea and occasionally dysentery in both children and adults [1]. EIEC strains are closely related to *Shigella* spp. AIEC is a recently emerged pathotype which has been associated with Crohn’s disease lesions [9,10].

ExPEC are frequently associated with nosocomial and community-associated infections. UPEC, distinct from the commensal *E. coli* strains in the phenotypic tracts and in virulence factors, is the most common microorganism of urinary tract infections (UTIs) in humans and is responsible for approximately 80% of the cases [1,11]. NMEC is a major cause of Gram-negative neonatal bacterial meningitis in developed countries with neurologic sequelae in many of the survivors [12]. In the last years, a significant increase in multidrug resistant NMEC strains has been observed [13]. APEC, an additional animal pathotype found in the intestinal microflora of healthy birds, is responsible for extraintestinal diseases in several avian species [14,15]. Recent studies have revealed that APEC and ExPEC have similarities in their serogroups and virulence factors suggesting a possible source of food-borne diseases [14,15]. Hovewer, the capacity of APEC to cause illness in human has not yet been verified [16].

**Table 1:** *E. coli* pathogenic types.

Pathotype (acronym)	Diseases	Symptoms	Virulence factors	Ref.
*Enteric E.coli*				
EnteroPathogenic *E. coli* (EPEC)	Diarrhoea in children	Watery diarrhoea and vomiting	Bfp, Intimin, LEE	[1]
EnteroHaemorrhagic *E. coli* (EHEC)	Haemorrhagic colitis, HUS	Bloody diarrhoea	Shiga toxins, Intimin, Bfp	[1,3]
EnteroToxigenic *E. coli* (ETEC)	Traveler’s diarrhoea	Watery diarrhoea and vomiting	Heat-labile and sheat-stable toxins, CFAs	[4,5]
EnteroAggregative *E. coli* (EAEC)	Diarrhoea in children	Diarrhoea with mucus and vomiting	AAFs, cytotoxins	[6,7]
Diffusely Adherent *E. coli* (DAEC)	Acute diarrhoea in children	Watery diarrhoea, recurring UTI	Daa, AIDA	[8]
EnteroInvasive *E. coli* (EIEC)	Shigellosis-like	Watery diarrhoea; dysentery	Shiga toxin, hemolysin, Cellular invasion, Ipa	[1,7]
Adherent Invasive *E. coli* (AIEC)	Associated with Crohn disease	Persistent intestinal inflammation	Type 1 fimbriae, Cellular invasion	[9,10]
*Extraintestinal E. coli (ExPEC)*				
UroPathogenic *E. coli* (UPEC)	Lower UTI and systemic infections	Cystitis, pyelonephritis	Type 1 and P fimbriae; AAFs, hemolysin	[1,11]
Neonatal Meningitis *E. coli* (NMEC)	Neonatal meningitis	Acute meningitis, sepsi	S fimbrie; K1 capsule	[12,13]
Avian Pathogenic *E. coli* (APEC)	Probable source of food-borne disease	-	Type 1 and P fimbriae; K1 capsule	[14,15]

Bfp: Bundle-forming pili; LEE: Locus for enterocyte effacement; HUS: haemolytic-uraemic syndrome; CFA: colonization factor antigen; AAF: aggregative adherence fimbria; Daa: diffuse adhesin; AIDA: adhesin involved in diffuse adherence; Ipa: Invasion plasmid antigen.

## 2. Mechanisms of Resistance

Antimicrobial resistance is a major and increasing global healthcare problem [17]. Since the introduction of the penicillin, a large number of bacteria have responded to the use of antibiotics with their ability to evolve and transmit antimicrobial resistance to other species [18]. Increased consumption of antimicrobial agents and their inappropriate use are among factors which further accelerated this phenomenon. Furthermore, the continuous migration of people between countries as well as international tourism and business travel play an important role in the acquisition and spread of multidrug resistant strains [19].

Antimicrobial resistance was also observed in animals, where the antimicrobials are used for therapy and prophylaxis of infectious diseases [20]. As in humans, the use of antimicrobials leads to an increased incidence of resistance in both pathogenic and endogenous bacteria [20]. Resistant bacteria from animals can infect humans by direct contact as well as via food products of animal origin. Multidrug resistance is defined as resistance to three or more antimicrobial classes to which bacteria do not show intrinsic resistance [21]. Multi-resistant strains are on the rise worldwide principally due to the spread of genes located on mobile genetic elements, including plasmids, integrons and transposons. Furthermore, the combination of these genes with chromosomally encoded resistance genes frequently results in bacteria that are resistant to all main classes of available antimicrobials [1,22].

*E.coli* is intrinsically resistant to therapeutic levels of penicillin G, the first β-lactam introduced into clinical practice, because of its outer membrane barrier. *E. coli* is also resistant to several different classes of antibiotics with distinct mechanisms of action [23,24]. However, this paper was restricted to β-lactams, quinolones and aminoglycosides, because the invasive *E. coli* isolated in the reporting countries were mainly resistant to their action [25]*.* Moreover, these molecules are defined “critically important antimicrobials” for human medicine [26].

In *E. coli*, β-lactamase production is the most important mediator of resistance to broad spectrum of β-lactams. β-lactamases constitute a wide class of enzymes, which are often encoded on plasmids, and are most commonly produced by *Enterobacteriaceae* in general and by *E. coli* in particular. β-lactamases confer resistance to penicillins and cephalosporins and are an emerging cause of multidrug resistance in Gram-negative bacteria. Several different types of β-lactamases have been described (Table 2) [27]. ESBLs confer resistance to several antibiotics including third- and fourth-generation cephalosporins and monobactams. The CTX-M-1 cluster is now the most prevalent type all over the world with the CTX-M-15 being the most identified variant [27,28,29,30,31,32,33,34]. In Europe, CTX-M-14 and CTX-M-15 types are widely distributed among humans [34]. Conversely, CTX-M-1 variant is the most prevalent among animals [34]. 

Carbapenem resistance in *Enterobacteriaceae* is a new emerging problem caused primarily by plasmid-encoded carbapenemases (Table 2) [25,35,36,37,38,39,40]. To date, these enzymes are mainly found in nosocomial isolates of *Klebsiella pneumoniae* and *E. coli* [36,38]. In Europe, carbapenemases-producing isolates are differently distributed among countries [35,36,37,38,39,40]. Furthermore, the prevalence of these strains appears to follow a north-south distribution [36,40].

*E. coli* also exhibits (fluoro)quinolone resistance which is frequently observed in conjunction with ESBL genes. Fluoroquinolone resistance in bacteria can be conferred by both chromosomal and plasmid-encoded genes (Table 2) [41,42,43,44]. Fluoroquinolone resistance *qnr* and *aac(6’)Ib-cr* genes have been frequently associated with β-lactam resistance genes, mainly *bla*_CTX-M-14_ and *bla*_CTX-M-15_ [32,45]. 

The aminoglycosides are bactericidal antibiotics, which act to inhibit protein synthesis, binding to the aminoacyl site of 16S rRNA within the bacterial 30S ribosomal subunits. There are several mechanisms that can cause bacterial resistance to aminoglycoside antibiotics (Table 2) [46,47,48,49]. In the last few years, alteration of 16S rRNA site by methyltransferase enzymes has emerged as a serious threat to this antimicrobial class. Of particular concern is 16S rRNA methyltransferase *armA* gene that confers pandrug-resistance to aminoglycosides and which is often accompanied by the carbapenemase genes on the same mobile genetic element [25,50,51].

**Table 2:** Mechanisms of resistance.

Mechanism	Example	Target	Ref.	
Enzyme inactivation	β-lactamases: TEM-type; SHV-type.	Broad-spectrum penicillins	[27]	
	ESBLs: TEM/SHV-type variants; Clusters: CTX-M-1, CTX-M-2, CTX-M-8, CTX-M-9, CTX-M-25; GES/PER/VEB types (less frequently).	Penicillins and cephalosporins	[27,28,29,30,31,32,33,34]	
	Class A: serine-carbapenemases; Class B: active zinc site metallo-β-lactamases; Class D: OXA β-lactamases.	Carbapenems	[35,36,37,38,39,40]	
Chromosomal mutations	Alterated target enzymes: DNA gyrase, topoisomerase IV. Decreased antimicrobial uptake: decrease in membrane permeability; overexpression of efflux pumps.	Quinolones	[41,42]	
Plasmid-mediated quinolone resistance	TMQRs: Qnr, AAC(6’)-Ib-cr), QepA, OqxAB.		[43,44]	
Enzyme inactivation	Acetyltransferases, nucleotidyltransferases, phosphotransferases.	Aminoglycosides	[46,47,48,49]	
Decreased antimicrobial uptake	decrease in membrane permeability; overexpression of efflux pumps.			
16S rRNA methylation	ArmA/Rmt family			

ESBLs: Extended spectrum β-lactamases; TMQRs: transferable mechanisms of quinolone resistance.

## 3. Epidemiology of Resistance

In Europe, antimicrobial resistance in Gram-negative bacteria is on the rise, particularly in *E. coli*, which constitutes a majority of invasive Gram-negative isolates in European countries [25,32,33,52,53,54,55,56,57,58,59]. The emergence and diffusion of multi-drug resistant strains of *E. coli* is complicating the treatment of several serious infections. *Enterobacteriaceae*, particularly *E. coli*, are the most frequent cause of hospital- and community-acquired infections [60,61,62].

Multidrug-resistant *E. coli* strains are also commonly isolated from animals and food products [34,63,64,65,66,67,68,69]. The use of antibiotics in animals contributed to the emergence and spread of the number of antibiotic-resistant strains, including *E. coli*, which can also infect humans through either direct contact with animals or through consumption of contaminated food [34]. *E. coli* is able to survive and adapt in various extraintestinal habitats and to spread resistances between humans, animals, their products and the environment through several transmission pathways [34].

Environment plays a key role in the spread of antimicrobial resistance serving as an unlimited reservoir of antimicrobial resistance genes [70]. Therefore, *E. coli* may acquire other drug resistance traits from environmental bacteria and conversely it can spread its resistance genes to potential pathogens in different habitats [71]. A source of multi-resistant *E. coli* could be represented by hospital effluents [72]. Several studies have reported the presence of multi-resistant strains in hospital effluents contributing to the spread of their antibiotic resistance genes in the municipal sewage systems and in the environment [72].

Antimicrobial resistant *E. coli* strains are distributed Europe-wide. The percentage of resistance to specific antibiotics in human sources varies substantially between countries—showing a north-to-south gradient—with the southern regions having the highest prevalence of resistance [25]. These geographical variations probably reflect differences in infection control practices and antibiotic use in the european countries. In Table 3 is presented the percentage (%R) of invasive strains, isolated from blood or cerebrospinal fluid, with resistance to three antimicrobials and with multi-resistance to all three antimicrobial classes. Data were analysed in accordance with the breakpoint criteria used by the local laboratory. The most widely used were EUCAST and CLSI breakpoints [25].

In the last annual surveillance report of the European Centre for Disease Prevention and Control (ECDC, data 2011), the presence of isolates resistant to third-generation cephalosporins, fluoroquinolones and aminoglycosides as well as isolates with resistance to all three antimicrobial classes was observed in all analyzed countries (27 EU Member States and three European Economic Area countries: Norway, Iceland and Liechtenstein) [25]. The percentage of isolates that express resistance to third-generation of cephalosporins is lowest in Sweden (3.0%), Norway (3.6%) and Finland (5.1%) and highest in Bulgaria (22.9%), Slovakia (31%) and Cyprus (36.2%). *E. coli* strains resistant to fluoroquinolones were present in low numbers in Sweden (7.9%), Norway (9.0%) and Estonia (9.9%) while they were predominant in Italy (40.5%), Slovakia (41.9%) and Cyprus (47.4%). Furthermore, the prevalence of isolates resistant to aminoglycosides ranged from 3.7% (Sweden) to 23.9% (Cyprus). The percentage is also higher in Romania (19.6%) Slovakia (17.9%) and Greece (16.8%). Finally, the prevalence of isolates with multi-resistance ranged from about 1% (*i.e.*, Estonia, Iceland and Sweden) to more than 10% (*i.e.*, Romania, Slovakia and Cyprus). Strains resistant to broad-spectrum penicillins were isolates in 28 countries, falling in the range of 34.8% (Sweden) to 77.6% (Cyprus). In contrast, only 0.04% of 59,326 isolates of *E. coli* were found to be resistant to carbapenems [25]. However, a recent survey reported that carbapenemase-producing *Enterobacteriaceae* continue to spread in Europe [40].

Antimicrobial resistance is also on the rise in European countries that were not included in the last annual ECDC report (Table 3) [19,57,59,66,73,74]. In a recent surveillance study conducted in 42 centres in Eastern Europe, about 15% of *E. coli* isolates were ESBL-positive and were collected in all countries participating in this project [59]. Turkey had the highest percentage with 25.2% of all ESBL-positive strains isolated [59]. Studies on fluoroquinolone resistance were also reported in Turkey [57,75]. Although prevalence of *qnr* genes among isolates is low, these strains exhibit a high level of resistance [68]. *E. coli* strains resistant to various antimicrobials were also isolated in Switzerland, Croatia and Bosnia and Herzegovina [57,74]. Fluoroquinolone resistance in *E. coli* was prevalent in these countries. Furthermore, multiresistant strains were also isolated, except in Bosnia and Herzegovina, probably due to limited surveillance data available for these countries [57]. ESBL-positive *E. coli* isolates resistant to quinolones and aminoglycosides were detected in Russia [37,45,56]. Resistance rates to ciprofloxacin and nalidixic acid in isolates were 12.9% and 17.2% respectively. Moreover, a lower percentage was observed for ESBL-positive isolates [45]. No data were found for other European countries, despite an extensive search.

**Table 3:** Antibiotic resistance of *E. coli* isolated (%R) from human sources in Europe.

Country	Third-generation cephalosporines	Fluoroquinolones	Aminoglycosides	Multi- resistance ^a^	Ref.
**Austria**	9.1	22.3	7.4	2.6	[25] ^b^
**Belgium**	6.0	21.5	9.3	1.4	[25]
**Bosnia and Herzegovina**	3.0	15.0	3.0	0	[57] ^c^
**Bulgaria**	22.9	30.2	17.3	10.1	[25]
**Croatia**	4.0	15.0	7.0	1.0	[57]
**Cyprus**	36.2	47.4	23.9	18.2	[25]
**Czech Republic**	11.4	23.5	8.8	3.7	[25]
**Denmark**	8.5	14.1	6.4	3.0	[25]
**Estonia**	12.2	9.9	4.8	1.1	[25]
**Finland**	5.1	10.8	5.3	2.7	[25]
**France**	8.2	17.9	7.9	2.6	[25]
**Germany**	8.0	23.7	7.6	3.6	[25]
**Greece**	14.9	26.6	16.8	10.8	[25]
**Hungary**	15.1	31.2	14.8	8.3	[25]
**Iceland**	6.2	14.0	6.2	0.8	[25]
**Ireland**	9.0	22.9	10.2	3.6	[25]
**Italy**	19.8	40.5	18.3	10.3	[25]
**Latvia**	15.9	16.8	11.4	9.2	[25]
**Lithuania**	7.0	12.9	9.7	2.4	[25]
**Luxembourg**	8.2	24.1	8.2	2.8	[25]
**Malta**	12.8	32.0	15.5	9.6	[25]
**Netherlands**	5.7	14.3	7.8	2.2	[25]
**Norway**	3.6	9.0	4.1	1.2	[25]
**Poland**	11.7	27.3	8.4	4.0	[25]
**Portugal**	11.3	27.2	16.1	7.5	[25]
**Romania**	22.0	30.4	19.6	10.9	[25]
**Slovakia**	31.0	41.9	17.9	12.9	[25]
**Slovenia**	8.8	20.7	9.8	4.1	[25]
**Spain**	12.0	34.5	14.8	4.9	[25]
**Sweden**	3.0	7.9	3.7	1.0	[25]
**Switzerland**	3.0	15.0	7.0	1.0	[57]
**Turkey**	42.0	52.0	35.0	23.0	[57]
**United Kingdom**	9.6	17.5	8.2	3.6	[25]

^a^ Isolates with resistance to all three antimicrobial classes; ^b^ data 2011; ^c^ data 2008.

## 4. *E. coli* Outbreaks

The epidemiology of *E. coli*—associated infections varies widely depending on the type of strain involved. In the last years in Europe, *E. coli* outbreaks were mainly caused by various EHEC strains.

STEC *E. coli* O104:H4 has been responsible for a large number of outbreaks in the recent years [3,76,77]. During the spring of 2011, a novel *E. coli* O104:H4 serotype infected about 4,000 individuals in Central Europe, mainly in Germany, provoking more than 900 cases of HUS [54,76]. This particular pathogen demonstrated a combination of virulence factors from both EAEC and EHEC strains [76]. A strain similar to the current outbreak strain had been previously isolated and characterised in Republic of Georgia [76].

HUS cases were reported in several European countries (Data 2010 [54]). The prevalent serogroups identified are O157 (EHEC O157:H7 serotype is the predominant cause of HUS) and O26. The highly virulent EHEC O26:H11/H^−^ serotype is emerging in Europe [78].

*E. coli* O25b:H4/ST131 (sequence type 131) is an emerging disseminated multidrug-resistant ExPEC strain, causing a broad spectrum of diseases, mainly urinary tract infections [31,65,79]. *E coli* O25b:H4/ST131 is widely distributed in Europe, with Spain and Italy most prominently affected [30,31,79,80].

## 5. Prevention and Control of *E. coli* Infections

In general, strategies for the prevention and control of the spread of *E. coli* should include access to safe water, good handling practices to reduce the risk of food contamination, sanitation measures, public education and vaccination [81,82,83].

Access to safe water is the primary target for the prevention of *E. coli* infections. Although this problem does not directly affect European countries, worldwide hundreds of millions people still do not have access to improved water sources (WHO/UNICEF Joint Monitoring Programme for Water Supply and Sanitation, 2012). Measures to prevent infections from food products include appropriate storage and cooking temperatures. Food irradiation technology may be used to drastically reduce bacterial load in high-risk products. Its use is authorised in European Union but limited to several products (EU Directive 1999/2/EC and 2009/C 283/02) However, food irradiation cannot be used as a substitute for hygiene and health practices or food good manufacturing or agricultural practice (EU Directive 1999/2/EC).

Hospital measures that limit risk of the spread of multiresistant pathogens include prevention of cross-contamination by implementing strict hygienic standard protocols as well as control over the use of antimicrobial drugs [81]. The main vehicles for pathogens’ spread are the hands of hospital workers and medical devices. Proper hand hygiene is critical for the prevention of cross-contamination.

Antibiotics are essential for the control and treatment of *E. coli* infections in humans and animals. However, it is generally accepted that antimicrobial resistance is associated with the quantity of antibiotic consumption [52]. The inappropriate use and misuse of antimicrobials increased the resistance in pathogens as well as in normal human bacterial flora in both. Animal reservoir is also an important source for resistance strains. Furthermore, the wide spread of antimicrobial therapy also results in the environmental release of antibiotics and antibiotic resistance genes with consequent selection of resistant bacteria. The effects of environmental release of the resistance genes are poorly studied. Antibiotic pollution promotes the fixation and mobilization of resistance genes between natural and clinical environments with world-wide spreading of resistance traits [84].

For this reason, a rational and responsible use of antibiotics should be a prerequisite for the prevention of the emergence and transmission of resistant bacteria [82]. Furthermore, appropriate strategies for monitoring and surveillance of the use of antibiotics are essential for the control and containment of the resistance, for the control of changes in bacterial populations, and for the development of suitable therapeutic strategies. Finally, greater attention should be given to the risks associated with release of antimicrobials into environment [84].

Probiotics could be an approach to the prophylaxis of several *E. coli* infections [85]. Probiotics are viable and safe microorganisms, principally belonging to the genera *Lactobacillus* and *Bifidobacterium*, which are able to colonize intestinal tract and thereby compete with pathogenic bacteria. Several studies on the potential use of probiotics for the prevention or therapy of gastrointestinal infections have been conducted. Treatment of infectious diarrhoea with probiotics demonstrated beneficial effects by reducing diarrhoea rates [86]. Studies on the effects of probiotics on inflammatory bowel diseases, including Crohn’s disease, also demonstrated beneficial effects, although results were modest [87]. The use of *Lactobacillus*, which is part of the microbiota in healthy humans, in the form of probiotics reduced the risk of UTI and vaginal infections [88].

Vaccination may be an important primary prevention strategy for human against the most harmful strains, such as ETEC, UPEC and NMEC. To date, no effective vaccine is available yet for the prevention of these infections.

Vaccine development against ETEC is still a global priority considering a high number of individuals infected both in developing countries and among travellers. Protective strategies against ETEC strains proved difficult to develop. However, several studies are in progress to obtain an effective vaccine that may have a substantial impact on children’s health in developing countries as well as protect travellers when visiting ETEC endemic areas [89,90]. In some countries, a cholera vaccine was used against ETEC strains to stimulate anti-heat-labile toxin immunity for short term protection [5].

About 80% of uncomplicated UTI are caused by UPEC, and the annual economic impact of this type of illness is very high, principally due to the costs associated with medical care and loss of productivity. Moreover, patient relapse after antibiotic therapy is not uncommon. All these considerations stimulate an ongoing search for the effective UPEC vaccine [91].

On the NMEC front, researchers implemented a non-conventional approach to vaccine development, research vaccinology [92]. This is an emerging genome-based vaccine development strategy that takes advantage of the complete repertoire of possible antigens that a bacterium can encode [83]. Preliminary data demonstrate that some antigens identified in NMEC, and combined with antigens of others ExPEC, could be used to develop a widely cross-reactive vaccine against ExPEC [92].

## 6. Alternative Therapies

The worldwide emergence of multidrug-resistant bacteria has dramatically limited the number of antibiotics that retain activity against these pathogens [93]. This problem has been further amplified by the dearth of novel classes of antibiotics. Therefore, development of novel therapeutic strategies for infectious diseases is high demand. In response, several new therapies have been developed, such as phage therapy, antimicrobial peptide therapy and combinations of two or more antibiotics [94,95,96,97].

The potential use of bacteriophages as therapeutic agents was recognized from the 1900s [95,98]. However, this therapeutic approach was eclipsed by the discovery and use of antibiotics. Nevertheless, phage therapy was used for the treatment of human bacterial infections, mainly in Eastern Europe [98,99]. Recently, the rise of multidrug-resistant bacteria and the consequent decrease in the number of effective antibiotics has forced scientists to search for alternative therapies [95]. Phages have a number of advantages that make them attractive for therapeutic use against bacteria [100]. First, they are highly specific and can be very effective in lysing bacteria. Second, phages are safe as underscored by several clinical studies, and third, they can be readily modified to fight the emergence of new multiresistant bacterial strains [100]. Many studies characterizing lytic phages specific for different *E. coli* strains have been published demonstrating their potential therapeutic value [13,101,102,103,104,105,106].

For example, phages are a promising therapeutic for UTIs caused by biofilm-forming UPEC strains. The majority of the UPEC strains produce biofilms which highly increase resistance to antibiotics. It has been observed that phages are able to pass through the extracellular matrix, to degrade the biofilm and kill bacteria [104,107,108]. A potential phage therapy was also demonstrated for sepsis and meningitis caused by NMEC multidrug resistant strain [13]. In experimental meningitis caused by a NMEC strain, the phage is able to cross the blood-cerebrospinal fluid barrier sterilizing the CSF [13].

Phages are also used to support food safety [105,109,110]. They are employed in food industry to prevent the contamination and bacterial proliferation on products, and to reduce bacterial charge during industrial food processing [105]. Phages are studied as disinfectants to control microbial contamination on food contact surfaces and equipment. Moreover, phage therapy was attempted to treat bacterial infections in animals [111,112].

In addition to therapeutic use of lytic phages, phage-encoded enzymes can be potentially used as an effective antibacterials against pathogens. Endolysins are hydrolase enzymes produced by phages at the end of their replication cycle to digest the bacterial cell wall for the release of progeny virions [113]. Endolysins work equally well when applied exogenously to bacterial cells and thus these enzymes are potentials candidates as new antibacterial agents [113,114].

The results obtained so far paint a positive picture for the future prospects of phage therapy against *E. coli* infections.

Antimicrobial peptides (AMPs) are an abundant and diverse group of molecules that are produced by eukaryotic and prokaryotic organisms or encoded by phages [96,97,115]. In eukaryotes, AMPs contribute to innate immune responses and defend organisms against potentially harmful microbes [97]. Bacteria use AMPs, referred to as bacteriocins, to kill other competitors in the same ecological niche. They are typically cationic amphipathic small peptides whose main mode of action is the ability to insert into membrane bilayers to form channels resulting in cell death [115]. Phage-encoded AMPs are a group of different lytic factors that allow for the release of viral progeny into the environment [97].

Several AMPs are being developed as drugs. They are able to act against antibiotic-resistant pathogens and are less susceptible to bacterial resistance than conventional antibiotics. Synthetic AMPs have been also developed, with designs based on common structural elements in natural peptides [96,116]. Synthetic AMPs are much more active than their native counterparts, less sensitive to proteases and have a low host toxicity profile.

Numerous natural and synthetic AMPs have direct activity against wide range of microorganisms including Gram-positive and Gram-negative. There are also several reports in the literature regarding activity of AMPs against *E. coli* strains [117,118,119,120]. Taken together, the results obtained so far highlight that AMPs represent a new promising therapeutic option for the treatment of bacterial diseases, including infections due to multidrug-resistant strains.

A novel approach to combating infections caused by multidrug resistant bacteria is combination therapy. The use of two or more antimicrobial agents simultaneously is a common practice for the treatment of several infectious, such as malaria, HIV and tuberculosis [94]. The use of combination therapy for the treatment of multidrug resistant bacteria, especially Gram-negative, could be an alternative to the development of new antibacterial drugs [121]. However, there is still considerable debate over the role of combination therapy *versus* monotherapy for Gram-negative infections. Although there are numerous reasons why combination therapy may be superior to monotherapy, the results obtained in clinical studies are not conclusive [121,122]. It has been suggested that combination therapy for the empirical treatment of severe Gram-negative infections, to be followed by transition to monotherapy once susceptibilities have been determined [122].

An alternative therapeutic strategy against multi-resistant bacteria could be the use of efflux pump inhibitors [123]. Efflux is a well known antibiotic resistance mechanism, bacteria being capable to export actively molecules from the cell using efflux pumps [124]. Although not used in the clinical practice yet, the high therapeutic potential of the combination of efflux pumps inhibitors with antibiotics has been clearly demonstrated [123]. Furthermore, this co-therapy would allow for the use of antibiotics normally compromised by efflux pump activity.

## 7. *E. coli* as a Biological Weapon

*E. coli* is present in the Centers for Disease Control and Prevention (CDC) list of biological agents potentially threat to public health and safety [125]. Several microorganisms or their products can be used as biological weapon for warfare and bioterrorism. The CDC classifies potential agents as biological weapon in three categories. In Category A agents which can be easily disseminated or spread from person to person, resulting in high mortality rate and impact on public health are listed. Category B lists pathogens moderately easy to disseminate, resulting in moderate morbidity rates and low mortality rates. Category C lists emerging pathogens with potentially high morbidity and mortality and which can be engineered for mass dissemination. *E. coli* O157:H7 strain is present in Category B as “food safety threat”. Even though less dangerous than Category A agents, Category B agents are easier to produce and handle, and the use of such agents against civilian populations by terrorists might well cause considerable panic [126].

## 8. Conclusions

Antimicrobial resistance in Europe continues to increase, markedly in Gram-negative bacteria, with considerable fluctuation between countries. In both humans and animals, the use of antimicrobials caused an increase in the incidence of resistance in both pathogenic and endogenous bacteria, highlighting a serious health problem to human medicine.

Information obtained from systematic surveillance studies is essential for monitoring changes in the antimicrobial resistance among pathogens, and for appropriate antibiotic treatments [127]. Therefore, to improve and enforce this network surveillance studies should be extended to all European countries. For this purpose, Central Asian and eastern European Surveillance on Antimicrobial Resistance (CAESAR) network has been established with the aim to expand surveillance to all european countries that are not part of the European Antimicrobial Resistance Surveillance Network (EARS-Net).
